# Identification of Key Off-Flavor Compounds in Thermally Treated Watermelon Juice via Gas Chromatography–Olfactometry–Mass Spectrometry, Aroma Recombination, and Omission Experiments

**DOI:** 10.3390/foods9020227

**Published:** 2020-02-20

**Authors:** Xiao Yang, Fan Yang, Ye Liu, Jian Li, Huan-Lu Song

**Affiliations:** Beijing Engineering and Technology Research Center of Food Additives, School of Food and Health, Beijing Technology and Business University, No. 11, Fucheng Road, Haidian District, Beijing 100048, China15733195507@163.com (F.Y.); lijian@th.btbu.edu.cn (J.L.); songhl@th.btbu.edu.cn (H.-L.S.)

**Keywords:** watermelon juice, thermal treatment, off-flavor, GC–O–MS, aroma recombination, omission

## Abstract

Thermally treated watermelon juice (TW) presents a strong unpleasant smell, resulting in poor consumer acceptance. It is necessary to identify the key off-flavor compounds in TW. Solid-phase microextraction (SPME) and solvent-assisted flavor evaporation (SAFE) coupled with gas chromatography–olfactometry–mass spectrometry (GC–O–MS) were applied to the extraction and analysis of the volatile compounds in TW. Five aroma-active compounds and seven off-flavor compounds were quantitatively analyzed by the standard curve method. Based on the flavor dilution factor (FD), odor attribute, odor activity value (OAV) of volatile compounds, and partial least-squares regression (PLSR) analysis, seven key off-flavor compounds were preliminarily identified as follows: (E)-2-heptenal, decanal, octanol, diisopropyl disulfide, hexanol, (E)-2-decenal, and (E)-2-octenol. Aroma recombination proved that these off-flavor compounds above had a negative impact on the overall flavor in TW. Omission experiments were taken to confirm them further. Finally, octanol, diisopropyl disulfide, and (E)-2-decenal were identified as the most potent off-flavor compounds in TW.

## 1. Introduction

Watermelon is a desirable fruit owing to its nutritional benefits. In China, the annual planting area and yield are approximately 1.85 million hm^2^ and 74.84 million tons, respectively, which accounts for 53.3% and 67.4% of the global production in 2017 [1]. Watermelon contains minerals, vitamins, and specific amino acids [2], and is especially rich in lycopene [3]. Consumption of lycopene-rich food may reduce the prevalence of certain types of cancers [4]. Hence, watermelon products have wide market potential.

Heating watermelon juice considerably affects its quality owing to its thermo-sensitive nature [5]. However, thermal treatment is a necessary step in industrial juice processing. The “steamed flavor” after thermal treatment affects consumer acceptance [6]. This considerably hinders the industrial processing of watermelon juice.

To identify the off-flavor compounds in thermally treated watermelon juice (TW), it is necessary to understand the mechanism of flavor change. Current studies have focused on the aroma of fresh watermelon (FW) or its juice. The flavor compounds in watermelon juice are mainly C6 and C9 aldehydes, ketones, and alcohols, such as nonanal, (E)-6-nonenol, (Z,Z)-2,6-nonadienal, (Z)-3-nonenal, (E,Z)-2,6-nonadienal, and (Z,Z)-3,6-nonadienal [7,8]. Development of off-flavor is an unavoidable issue in the thermal processing of watermelon juice; however, to the best of our knowledge, this is yet to be examined in TW. Investigations regarding off-flavors are mainly associated with other food materials. Dimethyl disulfide, dimethyl sulfide, dimethyl trisulfide, and 3-(methylthio) propanal contribute to the cooked flavor of melon juice during thermal processing [9]. The sulfur compounds and C5 aldehydes accumulate in winter melon juice during boiling [10]. Sensory experiments have shown that 2-methoxy-4-vinylphenol and dimethyl sulfide contribute to the typical stale off-flavor in stored orange juice (37 °C for four weeks) [11]. Hydrogen sulfide, dimethyl sulfide, methanethiol, and dimethyl trisulfide are important compounds contributing to cooked flavor in pasteurized milk [12].

In the present study, the aroma profiles of FW and TW were compared to identify the off-flavor compounds. The key off-flavor compounds in TW were determined using gas chromatography–olfactometry–mass spectrometry (GC–O–MS), odor attributes, and partial least-squares regression (PLSR) analysis. Furthermore, aroma recombination and omission experiments were performed to verify the key off-flavor compounds.

## 2. Materials and Methods

### 2.1. Chemicals

n-Alkanes (C7-C30) for the retention index (RI) calculation, 2-methyl-3-heptanone (99%, internal standard), (E,Z)-2,6-nonadienal (95%), (E)-2-heptenal (97%), decanal (≥98%), octanol (≥99%), (E,Z)-2,6-nonadienol (≥95%), (E)-2-octenal (≥95%), diisopropyl disulfide (≥96%), hexanol (≥99%), (E)-2-nonenal (97%), (E)-2-decenal (≥95%), nonanol (98%), and (E)-2-octenol (97%) were purchased from Sigma-Aldrich (Beijing, China). Hexane, diethyl ether, n-pentane, and anhydrous Na_2_SO_4_ used for extraction and separation of flavor substances were all analytical reagent and provided by Banxia Scientific Instruments Co. Ltd. (Beijing, China).

### 2.2. Preparation of Samples

Qilin is the main cultivar with a large yield in China. Twenty watermelons (cultivar: Qilin, seedless, weighing approx. 0.4 kg each; locality of growth: Panggezhuang Town, Daxing District, Beijing) were one-time selected and purchased from Beijing Yonghui Supermarket randomly on June 10th, 2018. The pulp was blended (Philips, HR2860, Zhuhai, China), and quickly filtered through a nylon mesh (200 mesh). FW was analyzed immediately. Batches of 100 mL juice were vacuum-packed with an odorless vacuum packaging bag with aluminum foil immediately and frozen using liquid nitrogen. All the packed juices (100 mL for each bag) were stored at −80 °C for further analysis. TW was processed using a water bath at 70 °C for 20 min according to the pasteurization method (the juice flavor changed greatly under this condition through the pre-experiments. A thermometer was inserted into the juice through the bag and timing began when the center temperature of juice reached 70 °C) [13].

### 2.3. Extraction of Flavor Compounds From Watermelon Juice by Solid-Phase Microextraction (SPME)

Volatile organic compounds of a 10 mL sample of watermelon juice with 1 μL internal standard (2-methyl-3-heptanone, 0.816 μg/μL) added in a 40 mL headspace vial (Agilent, Inc., Santa Clara, CA, USA) were extracted manually with SPME using a 2 cm, 50/30 μm divinylbenzene/carboxen/polydimethylsiloxane fiber (Supelco, Inc., Bellefonte, PA, USA). Samples were allowed to equilibrate for 20 min at 40 °C before collection for 40 min with continuous stirring at 100 rpm (J&K Scientific Ltd., Beijing, China) [14].

### 2.4. Extraction of Flavor Compounds From Watermelon Juice by Solvent-Assisted Flavor Evaporation (SAFE)

One hundred milliliters of watermelon juice was mixed with 150 mL diethyl ether-pentane mixture (2:1, *v*/*v*) and stirred for 8 h. Fifty microliters of 2-methyl-3-heptanone (0.816 μg/μL) were added as the internal standard. Then, volatiles were extracted from the solvent extracts by distillation for 2 h at 10^−4^ torr. The solvent layer was concentrated to 2 mL with a Vigreux column (Heqi Glass Instrument Co., Ltd, Shanghai, China) after being dried through an anhydrous Na_2_SO_4_ column. The volume further reduced to 0.2 mL under a flow of nitrogen [15].

### 2.5. GC–O–MS Analysis

GC–MS (7890A-7000B; Agilent Technologies Inc., Santa Clara, CA, USA) with an olfactometer (Sniffer 9000; Brechühler, Schlieren, Switzerland) was used to analyze the volatile compounds. DB-WAX and DB-5 chromatographic columns (30 m × 0.25 mm × 0.25 μm; J&W Scientific, Folsom, CA, USA) were employed to separate these compounds [14].

Mass spectra in electron ionization mode were recorded at 70 eV and a mass/charge range of 50–350 amu at a 2.0 scan s^−1^ scan rate. Compounds were identified according to NIST 14.0 (The National Institute of Standards and Technology, Gaithersburg, MD, USA) mass spectra libraries.

GC–O analysis was carried out on polar and non-polar columns by three well-trained panelists. Before analysis, the panelists were trained by smelling the odors of the model solutions of reference compounds at different concentrations. The aroma descriptor, intensity value, and retention time were recorded by the panelists during analysis [15]. If two or more panelists detected the aroma, an aroma-active location was identified.

### 2.6. Identification of Key Flavor Compounds

Two types of dilution analysis were used to identify key flavor compounds, including headspace dilution analysis (HDA) for SPME and aroma extraction dilution analysis (AEDA) for SAFE, as described by Zhang et al. [15]. For SPME (Supelco, Inc., Bellefonte, PA, USA), the volatile compounds were diluted stepwise by increasing the split ratio of 1:3. For SAFE (Glasbläserei Wolfgang Bahr, Manching, Germany), the concentrated fraction was diluted stepwise at the ratio of 1:3 with a diethyl ether-pentane mixture (2:1, *v*/*v*). The process was ceased when aromas could not be smelled. FD factor could be expressed as the ratio of the initial and final concentration of the flavor compound of juice. The compounds with FD higher than 1 were identified as key flavor compounds.

### 2.7. Qualitative Analysis of Flavor Compounds

The compound identification was carried out by NIST 14.0 mass spectrum database, the retention index (RI), and odor properties. The key flavor compounds were confirmed further compared with the standard compounds. RI was calculated using Equation (1) and compared with the references.

(1) $$RI=100n+100\frac{t_{a}-t_{n}}{t_{n+1}-t_{n}}$$

where t_a is the retention time of the sample “a”, t_n is the retention time of Cn, “n” represents the number of carbon atoms, and the retention time of sample “a” is between Cn and Cn+1 [16].

### 2.8. Quantitative Analysis of Flavor Compounds

GC conditions were the same as mentioned above, and a selected ion monitor (SIM) was selected as the mass acquisition mode. Both extract methods were used to quantify different ions. Reference standards with a series of concentrations were prepared. The mixed reference standards (1 μL) were added to the sample gathered with internal standard (1 μL, 0.816 μg/μL). The extraction procedures were the same as the methods of SPME and SAFE as described above. Standard curves were established based on the peak area and concentration of each compound. The *Y*-axis represented the peak area ratio of analyte to the internal standard, and the *X*-axis represented the concentration of reference standards of the analyte [17]. In order to eliminate the loss during the extract process, the recovery of the target compound was calculated using Equation (2).

(2) $$Recovery\left(\%\right)=\frac{\left(C_{1}-C_{0}\right)}{C_{2}}\times 100\%$$

where *C*_0_ is the concentration of the compound before reference standard being added, *C*_1_ is the detected concentration after reference standard being added, and *C*_2_ is the reference standard concentration being added [16].

### 2.9. Odor Activity Value (OAV)

The equation to calculate OAV was as below:

(3) $$OAV=\frac{C_{i}}{OT_{i}}$$

where *C_i_* is a compound concentration and *OT_i_* is the odor threshold of this compound. Compounds with OAV ≥1 were considered to contribute to the juice flavor [18].

### 2.10. Sensory Evaluation

Twelve panelists (6 males and 6 females, aged 20–35 years) were recruited from the Molecule Sensory Laboratory of Beijing Technology and Business University. Members of the sensory panel were trained for 2 months to familiarize the watermelon aroma characteristics. Sensory evaluation was strictly in accordance with Table 1. The total score was based on the 5-point scale, with 0 for no odor and 5 for the strongest odor. Each sample was evaluated three times by every panelist to make sure that the score differences were no more than 20%.

### 2.11. Aroma Recombination of TW

To verify the obtained result of off-flavor compounds, the aroma recombination system was prepared and compared with the actual watermelon juice flavor [19]. A model aroma mixture system was prepared in ultrapure water containing 5% fructose and citric acid, 0.3% pectin, and reference standards of the five aroma-active compounds and seven key off-flavor compounds at the concentration were quantified. The total soluble solid of the model mixture was adjusted to 8.00 ± 0.06 Brix, and pH 5.70 ± 0.10. The flavor similarity between the aroma recombination system and TW was compared based on the score rules of sensory evaluation listed in Table 1. Every sensory evaluation was conducted in triplicate.

### 2.12. Omission Experiments

Mixture models were produced by omitting one kind of key off-flavor compounds from the aroma recombination system. The same sensory panels evaluated the flavor similarity between the omission and recombination models in a triangle test. Every sensory evaluation was conducted in triplicate [19].

### 2.13. Statistical Analysis

Analysis of variance was carried out to determine the significance at a 95% confidence interval using SAS 9.3 software (Statistical Analysis System, Cary, NC, USA). Partial least-squares regression (PLSR) was implemented using SIMCA-P 11.5 software (Umetrics, Umeå, Sweden). All experiments were performed in triplicate.

## 3. Results and Discussion

### 3.1. Identification of Aroma-Active Compounds in FW and TW

In order to extract and analyze the flavor compounds of watermelon juice comprehensively, the extract methods of SPME (non-solvent extract) and SAFE (solvent extract), the chromatographic columns of DB-Wax (strong polar) and DB-5 (weak polar) (Agilent Technologies Inc., Santa Clara, CA, USA) were applied in this study. Thus, the aroma-active compounds and key off-flavor compounds could be identified accurately and thoroughly. Fifty-seven compounds in FW and TW were extracted and identified (Table 2). Five groups of flavor compounds were identified in FW and TW, containing 26 aldehydes, 11 alcohols, 7 ketones, 5 sulfur compounds, and 8 others. Table 2 shows the presence of 17 compounds in total with FD >1 (7, 9–11, 13, 14, 16, 17, 27, 29, 33–36, 39, 47, 48) by GC–O–AEDA of the isolates in FW and TW. Among the 17 detected compounds, 13 odorants (7, 9–11, 13, 14, 17, 27, 29, 34, 36, 39, 47) were present in both kinds of juices. Four compounds, including (E)-2-decenal, (E)-2-octenol, (E,Z)-3,6-nonadienol, and dipropyl trisulfide were only detected in TW, which contributed to the off-flavor of TW.

#### 3.1.1. Aldehydes

Among the 17 compounds identified in FW and TW, aldehydes were the most prevalent and potent aroma compounds in watermelon or its juice [7,8]. Of the 26 aldehydes, eight (7, 9–11, 13, 14, 16, 17) were the aroma-active compounds with FD >1. These aldehydes were responsible for imparting the soapy, fatty, or green smells. C6 and C9 unsaturated aldehydes are derived from polyunsaturated fatty acids (PUFA), such as linoleic acid and linolenic acid [7]. 9, 13-Hydroperoxides are primary PUFA oxidation products in plants. Herein, its decomposition by enzymes generated multiple fruity like aldehyde derivatives with shorter chains [7], such as (E)-2-nonenal, (E,Z)-2,6-nonadienal, (E)-2-decenal, and (E,E)-2,4-nonadienal. C9 unsaturated aldehydes are considered to contribute to melon aroma greatly due to their low odor thresholds [14]. Among melons, (E,E)-2,4-heptadienal and (E)-2-heptenal are reported in seedless watermelon and those from Ibaraki, Japan [20]. They are also present in the avocado, fish oil, and olive oil. These aldehydes derive from the oxidation of polyunsaturated fatty acids [21,22]. In these studies, (E)-2-heptenal imparts fatty, fruity, or green smell, and (E,E)-2,4-heptadienal contributes a green, fatty, or nutty note, which are in accordance with this study. (E)-2-Octenal was detected in grafted and mini-watermelon, and three cultivars of muskmelons [8,20,23,24]. Decanal was detected in Jiashi muskmelon, mini-watermelon, and heated soybean oil [8,20,25,26]. It easily contributes to subsequent reactions during the thermal treatment as a keto–enol tautomerism product of the combination product between 1-decenyl radical decomposed from O-8-hydroperoxide and hydroxyl radical [26]. Specifically, (E)-2-decenal only exists in TW (FD = 27) with a mechanical and soapy smell. This contributed to the strong off-flavor of TW.

#### 3.1.2. Alcohols

Eleven alcohols, six of which (27, 29, 33-36) were identified with FD >1 in both FW and TW. Hexanol, octanol, (E)-2-octenol, nonanol, (E,Z)-3,6-nonadienol, and (E,Z)-2,6-nonadienol could be formed due to reduction of the corresponding aldehydes. All identified compounds were previously reported for watermelon aroma, and presented fatty, fruity, floral, green, or cucumber-like smell [8,23]. Among them, (E)-2-octenol and (E,Z)-3,6-nonadienol were only detected in TW (FD = 3 and 3) with plastic, soapy, or fishy smell slightly contributing to the off-flavor.

#### 3.1.3. Ketones

Among the seven identified ketones, geranyl acetone was found with FD >1 in both FW and TW. They were reported in the melon fruits with different rootstocks, seedless watermelon, and watermelon juice treated by high-intensity pulsed electric fields [6,14,23]. Geranyl acetone probably derived from phytoene or phytofluene. In essence, color is highly associated with aroma compounds in watermelon, and this relationship is probably a function of the degradation of carotenoids into volatiles [27]. Moreover, its FD increased from three to nine, indicating that TW had a stronger floral or green smell than FW.

#### 3.1.4. Sulfides

Five sulfides, diisopropyl disulfide, and dipropyl trisulfide were identified with FD >1 both in FW and TW. Few reports have described sulfides in watermelon or its juice, although these compounds exist in Jiashi muskmelon [25]. These two compounds were detected in our previous study, and extracted only by SAFE [28]. A strong possibility exists that the sulfur compounds may originate from methionine present in the watermelon seeds [29]. Sulfides presented the flavor characteristic of onion or garlic. They impacted the flavor quality of watermelon juice. Moreover, dipropyl trisulfide was only detected in TW (FD = 3) with vegetables or garlic smell, suggesting that it slightly contributes to the off-flavor.

As shown in Table 2, based on FDs, the aroma-active compounds in FW and TW are as follows: (E,Z)-2,6-nonadienal (FD >81/>81), (E)-2-octenal (FD = 27/>81), (E,Z)-2,6-nonadienol (FD = 27/81), (E)-2-nonenal (FD = 27/27), nonanol (FD = 27/27). The results were in accordance with previous reports [28].

### 3.2. Identification of Key Off-Flavor Compounds in TW

Thermal treatment promoted the formation and release of flavor compounds with higher FDs in TW. After thermal treatment, watermelon juice produced a strong unpleasant flavor. As shown in Table 2, FDs of some compounds increased significantly after thermal treatment, including (E)-2-heptenal (fatty, fruity, green), (E)-2-octenal (fatty, nut), (E,E)-2,4-heptadienal (nut, fatty), (E)-2-nonenal (cucumber, green), (E,Z)-2,6-nonadienal (cucumber, green), (E)-2-decenal (mechanical, soapy), octanol (metal, burnt), (E)-2-octenol (plastic, soapy), (E,Z)-3,6-nonadienol (fishy), (E,Z)-2,6-nonadienol (cucumber), geranyl acetone (floral, green), (Z)-β-ionone (oat, floral), and diisopropyl disulfide (garlic, sulfur) [15]. Through the combination of FD values and odor characteristics, the compounds with unpleasant odor characteristics, and the FDs in TW being equal to or higher than that in FW, were identified as the off-flavor compounds. Thus, seven compounds were preliminary considered to be key off-flavors in TW: (E)-2-heptenal (fatty, fruity, green; FD = 81), decanal (pungent, soapy; FD = 81), octanol (metal, burnt; FD = 81), diisopropyl disulfide (garlic, sulfur; FD = 81), hexanol (bitter, floral; FD = 81), (E)-2-decenal (mechanical, soapy; FD = 27), and (E)-2-octenol (plastic, soapy; FD = 3).

**Figure 1:**
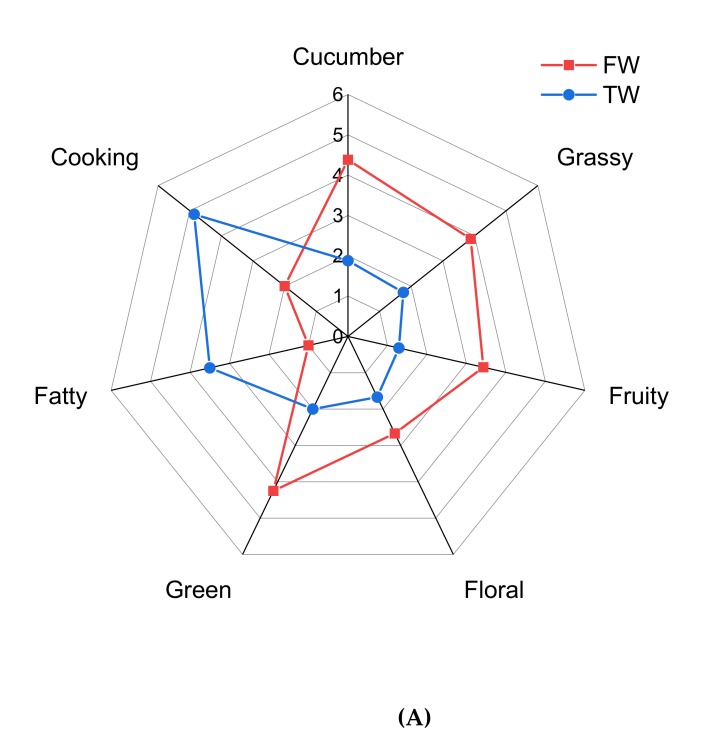

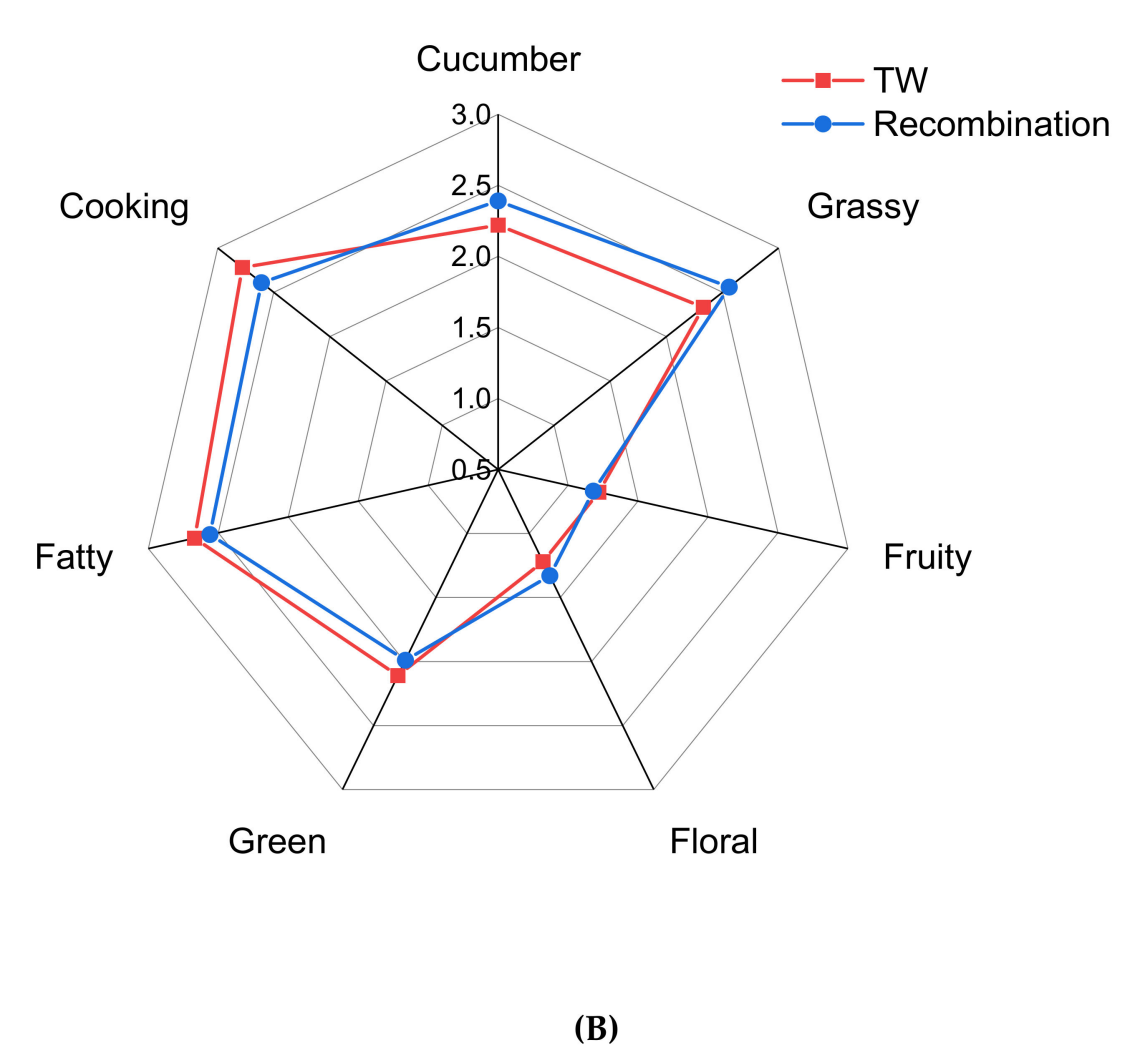
Odor attributes of FW and TW (**A**) and aroma profile of TW and recombination system (**B**) by sensory evaluation.

As shown in Figure 1A, seven odor attributes were generally summarized in FW and TW. A pleasant odor, such as “cucumber”, “grass”, and “green” had higher scores in FW. While the scores of unpleasant odor, such as “cooking flavor” and “fatty”, increased in TW significantly. This result was related to FDs increase in the following off-flavor compounds (Table 2): (E)-2-heptenal (fatty, fruity, green), (E)-2-decenal (mechanical, soapy), octanol (metal, burnt), (E)-2-octenol (plastic, soapy), and diisopropyl disulfide (garlic, sulfur). The off-flavor of TW was caused by the combination of multiple above compounds [30]. In thermally processed muskmelon juice, volatile sulfur compounds, and small molecular aliphatic aldehydes also contributed to the off-odors [31]. Methanethiol and dimethyl sulfide were the key off-flavor compounds identified in thermal mandarin juices [32]. Thermal degradation of sulfur-containing amino acids and unsaturated fatty acid, as well as Maillard reactions in juice, contributed to the generation of the thermal-induced off-notes [33,34].

**Figure 2:**
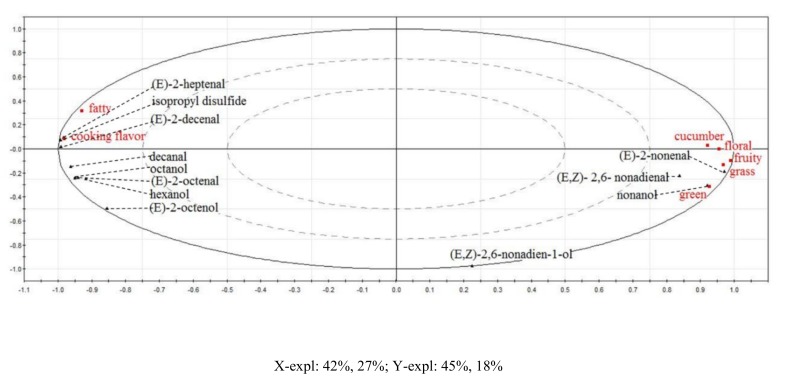
Correlationship analysis of odor attributes and aroma-active compounds in FW and TW by partial least-squares regression (PLSR).

As shown in Figure 2, 7 flavor attributes and 12 compounds (aroma-active compounds and key off-flavor compounds) in FW and TW were selected to conduct PLSR analysis, they were taken as variable X and Y, respectively. The PLSR model provided a two-factor model explaining 69% of the X-variance (flavor attributes) and 63% of the Y-variance (aroma-active compounds and key off-flavor compounds) in Figure 2.

The odor attributes of “fatty” and “cooking flavor” concentrated on the left side of the loading plot, which presented significant positive correlations with compounds: (E)-2-heptenal, diisopropyl disulfide, (E)-2-decenal, decanal, (E)-2-octenol, octanol, and hexanol (*p* < 0.05). In summary, these seven volatile compounds were preliminarily identified as the key off-flavor compounds in TW.

### 3.3. OAV of Aroma-Active and Off-Flavor Compounds

Three quantitative ions were selected in order to correctly match with known compounds from the quality library. The recovery (70–130%) was calculated to guarantee the accuracy of the quantitative results (Table 3). The standard curves possess good linearity with correlation coefficient: *R*^2^ ≥ 0.99. All data displayed improved repeatability with RSD ≤10%. The concentration of five aroma-active compounds and seven off-flavor compounds are shown in Table 3. Among them, diisopropyl disulfide had the highest concentration (2122.17 μg/μL), followed by (E)-2-heptenal (1090.23 μg/μL) and (E,Z)-2,6-nonadienol (825.18 μg/μL).

OAV was another index that contributed aroma compounds to the overall flavor [18]. As shown in Table 4, the highest OAV in TW was diisopropyl disulfide (OAV = 21,222), followed by (E)-2-nonenal (OAV = 3146) and (E,Z)-2,6-nonadienal (OAV = 656). Ten compounds with OAV ≥1 contributed to the overall flavor of watermelon juice, which was in accordance with FDs. However, hexanol and (E)-2-octenol had higher FDs (81 and >81) and lower OAV (OAV <1) due to lower concentration or higher threshold. In the real food matrix, different odor compounds interact due to the antagonistic and synergistic effect, not a simple superposition [35]. Individual differences were completely unavoidable, including perception and cognition of flavor compounds. For OAV, every compound had the same psychometric function and aroma intensity, which increased linearly with increasing concentration [36]. However, the results contradicted the real matrix. The relationship between compound concentration and its response was not linear but S-shaped [24]. Hence, the identification of the key off-flavor compounds needed further verification.

### 3.4. Aroma Recombination of TW

According to the quantitative results of TW, the aroma recombination was carried out to verify the contribution of the seven key off-flavor compounds to the overall flavor of TW. As shown in Figure 1B, the aroma recombination system performance displayed good similarity with TW, with no significant difference between the seven odor attributes (*p* < 0.05) being observed. This indicated that the identification and quantitation experiments were accurate, and that the aroma-active and key off-flavor compounds were precisely identified [14,43]. Therefore, (E)-2-heptenal, diisopropyl disulfide, (E)-2-decenal, decanal, (E)-2-octenol, octanol, and hexanol were confirmed as the key off-flavor compounds in TW.

### 3.5. Omission Experiments

In order to further verify and rank the contribution level among the seven key off-flavor compounds, omission experiments were divided into seven groups (Table 5). When octanol was omitted, 12 sensory panelists judged the flavor difference correctly from three samples, which showed the highest significant difference (*p* ≤ 0.001). This result revealed that octanol played a very important role in the overall flavor in TW. Among them, the absence of diisopropyl disulfide and (E)-2-decenal showed significant differences (*p* ≤ 0.05 and *p* ≤ 0.01), which agreed with the higher FDs and OAVs, respectively. Therefore, these two compounds also had a significant influence on the overall flavor of TW. The omission experiments: hexanol and (E)-2-octenol (OAV ≤1 but higher FDs) also showed significance differences (*p* ≤ 0.01 and *p* ≤ 0.05). However, no significant differences were observed when (E)-2-heptenal and decanal were omitted from the recombination in spite of their higher FDs and OAVs. Comprehensive consideration of significance difference of omission experiment, FD, and OAV, octanol (metal, burnt; FD = 81; OAV = 2), diisopropyl disulfide (garlic, sulfur; FD = 81; OAV = 21,222), and (E)-2-decenal (mechanical, soapy; FD = 27; OAV = 294) were identified as the most potent off-flavor compounds in TW.

The above three compounds were previously reported as off-flavor compounds. Octanol contributes to the off-flavor of whey protein concentrate during storage of 45 °C for 15 weeks, and it changes the organoleptic properties of packaged food [44,45]. In bovine bone marrow extract, it contributes to the off-flavor due to the Maillard reaction [46]. From this aspect, the Maillard reaction might also induce the off-flavor. (E)-2-decenal was employed as an aroma marker of oxidation degradation to quantitatively monitor and describe the quality of packaged olive oil [47]. (E)-2-Decenal also formed by oleic acid being degraded at 140 °C affects the flavor of frying [48,49]. It is derived from the thermal reaction of enzymatic hydrolysates of the protein with oxidized lard [50]. Diisopropyl disulfide showed a positive relationship with the sensory attributes “salt flavor” and “carrot aroma” in the commercial regular salt soup, and also a “sulfury, onion” note in the preserved egg yolk [15,51]. These results are in accordance with this study.

## 4. Conclusions

In conclusion, seven key off-flavor compounds in TW were preliminarily identified by concentration variation, odor attributes, and PLSR analysis. Five aroma-active compounds and seven key off-flavor compounds were quantified by the standard curve method. They were further confirmed by both OAV and FD. The aroma recombination was employed to verify the contribution of the seven key off-flavor compounds to the overall aroma profile. In addition, the omission experiment from the recombination system was carried out to confirm the results. Octanol, diisopropyl disulfide, and (E)-2-decenal were identified as the most potent off-flavor compounds in TW.

## Figures and Tables

**Table 1:** Flavor attributes selected for sensory evaluation.

Flavor Attributes	Characteristic
cucumber	fresh cucumber
grass	chopped freshly grass
fruity	mixed aroma associated with fresh fruit
floral	light aroma associated with fresh flowers
fatty	oily aroma like plant oils or animal fats
cooking	cooking smell with high temperature
green	pleasant aroma of fresh plant

**Table 2:** Flavor compounds identified by gas chromatography–olfactometry–mass spectrometry (GC–O–MS) in fresh watermelon (FW) and thermally treated watermelon juice (TW) extracted by solid-phase microextraction (SPME) and solvent-assisted flavor evaporation (SAFE).

Categories	Compounds ^a^	CAS	Odor Property	RI ^b^		Identification Methods ^c^	FD ^d^		Extraction Methods
				DB-WAX	DB-5		**FW**	**TW**	
**Aldehydes (26)**									
1	2-methylbutanal	96-17-3	cocoa, almond	964	692	MS,RI,S	-	-	SPME, SAFE
2	hexanal	66-25-1	grass	1068	794	MS,RI,S	-	-	SPME, SAFE
3	(*E*)-2-pentenal ^#^	1576-87-0	strawberry, fruity	1117	746	MS,RI	-	-	SPME
4	heptanal	111-71-7	fatty, putrid	1174	897	MS,RI,S	-	-	SPME
5	(*E*)-2-hexenal ^#^	6728-26-3	green, fruity	1207	847	MS,RI,S	-	-	SPME
6	octanal	124-13-0	pungent, soapy	1280	998	MS,RI,S	-	-	SPME, SAFE
7	(*E*)-2-heptenal *	18829-55-5	fatty, fruity, green	1314	952	MS,RI,O,S	27	81	SPME
8	nonanal	124-19-6	green, fatty	1383	1100	MS,RI,S	-	-	SPME, SAFE
9	(*E*)-2-octenal *	2548-87-0	fatty, nut	1420	1054	MS,RI,O,S	27	>81	SPME, SAFE
10	(*E,E*)-2,4-heptadienal *	4313-03-5	nut, fatty	1482	-	MS,RI,O,S	3	3	SPME
11	decanal	112-31-2	pungent, soapy	1490	1202	MS,RI,O,S	81	81	SPME
12	benzaldehyde	100-52-7	almond, caramel	1509	957	MS,RI,S	-	-	SPME, SAFE
13	(*E*)-2-nonenal *	18829-56-6	cucumber, green	1527	-	MS,RI,O,S	27	27	SPME, SAFE
14	(*E,Z*)-2,6-nonadienal *	557-48-2	cucumber, green	1576	-	MS,RI,O,S	>81	>81	SPME
15	β-cyclocitral	432-25-7	mint	1614	1222	MS,RI	-	-	SPME
16	(*E*)-2-decenal *	3913-81-3	mechanical, soapy	1635	1259	MS,RI,O,S	-	27	SPME
17	(*E,E*)-2,4-nonadienal	5910-87-2	fatty, green	1691	1192	MS,RI,O,S	9	9	SPME
18	citral	5392-40-5	lemon	1726	1268	MS,RI	-	-	SPME
19	2,6-dimethyl-5-heptenal	106-72-9	fruity	-	1050	MS,RI	-	-	SPME
20	(*Z*)-4-heptenal	6728-31-0	cream	-	892	MS,RI	-	-	SPME
21	2-undecenal	2463-77-6	sweet	-	1346	MS,RI	-	-	SPME
22	undecanal	112-44-7	fatty, sweet	-	1303	MS,RI	-	-	SPME, SAFE
23	(*E,E*)-2,4-nonadienal	25152-84-5	fried	1802	1314	MS,RI,S	-	-	SPME
24	acetal	105-57-7	fruity	900	722	MS,RI	-	-	SAFE
26	dodecanal	112-54-9	floral	-	1405	MS,RI	-	-	SAFE
**Alcohols (11)**									
27	hexanol	111-27-3	bitter, floral	1346	865	MS,RI,O,S	81	81	SPME, SAFE
28	(*Z*)-3-hexenol	928-96-1	grass	1376	852	MS,RI	-	-	SPME, SAFE
29	Octanol *	111-87-5	metal, burnt	1548	1068	MS,RI,O,S	81	81	SPME
30	heptanol	111-70-6	green	-	967	MS,RI,S	-	-	SPME
31	1-octene-3-ol	3391-86-4	mushroom	1394	977	MS,RI,S	-	-	SPME
32	benzyl alcohol	100-51-6	floral	1865	1037	MS,RI	-	-	SPME, SAFE
33	(*E*)-2-octenol *	18409-17-1	plastic, soapy	1601	1167	MS,RI,O,S	-	3	SPME
34	nonanol	143-08-8	fatty, green	1650	1170	MS,RI,O,S	27	27	SPME, SAFE
35	(*E,Z*)-3,6-nonadienol *	56805-23-3	fishy	1738	-	MS,RI,O,S	-	3	SPME, SAFE
36	(*E,Z*)-2,6-nonadienol *	28069-72-9	cucumber	1754	-	MS,RI,O,S	27	81	SPME, SAFE
37	2-methylbutanol	137-32-6	wine	-	745	MS,RI	-	-	SAFE
**Ketones (7)**									
38	6-methyl-5-hepten-2-one	110-93-0	rubbery	1327	983	MS,RI	-	-	SPME, SAFE
39	geranyl acetone *	3796-70-1	floral, green	1844	1450	MS,RI,O,S	3	9	SPME, SAFE
40	(*Z*)-*β*-ionone *	79-77-6	oat, floral	1931	-	MS,RI,O,S			SPME
41	3-octanone	106-68-3	medicine, fatty	1248	-	MS,RI	-	-	SPME
42	2-butanone	78-93-3	floral	894	-	MS,RI	-	-	SAFE
43	2-pentanone	107-87-9	fruity	-	684	MS,RI,S	-	-	SAFE
44	2-hexanone	591-78-6	ether	-	729	MS,RI	-	-	SAFE
**Sulfides (5)**									
45	diethyl disulfide	110-81-6	pungent, garlic	1206	-	MS,RI	-	-	SAFE
46	ethyl propyl disulfide	30453-31-7	garlic	1231	970	MS,RI	-	-	SAFE
47	diisopropyl disulfide *	4253–89-8	garlic, sulfur	1249	1016	MS,RI,O,S	81	81	SAFE
48	dipropyl trisulfide	6028-61-1	vegetable, garlic	1527	1231	MS,RI,O,S	-	3	SAFE
49	methyl propyl disulfide	2179-60-4	sulfur, garlic	1112	-	MS,RI	-	-	SAFE
**Others (8)**									
50	2-n-pentylfuran	3777-69-3	fatty	1220	988	MS,RI,S	-	-	SPME
51	ethyl acetate	141-78-6	fruity	884	-	MS,RI	-	-	SAFE
52	o-xylene	95-47-6	floral	1126	865	MS,RI	-	-	SPME, SAFE
53	meta-xylene	108-38-3	plastic	1119	856	MS,RI	-	-	SPME, SAFE
54	limonene	5989-27-5	green, fruity	1194	-	MS,RI	-	-	SAFE
55	naphthalene	91-20-3	wax	1733	1185	MS,RI	-	-	SAFE
56	styrene	100-42-5	gasoline	-	886	MS,RI	-	-	SAFE
57	3-methylbutyric acid	503-74-2	sweat	-	909	MS,RI,S	-	-	SAFE

a Compounds marked with “*” mean there were significant differences in the concentration between FW and TW; “#” means the compounds were only detected in TW; b compounds were separated respectively by DB-WAX and DB-5 columns; the actual RI could not exceed ±50 of the library standard value; compounds marked with “-” means they were not detected; c MS, compounds were identified by MS spectra; O, compounds were identified by sniffing; RI, compounds were identified by comparison to reference standards; d compounds marked with “-”, which means the compound could not be identified by sniffing.

**Table 3:** Standard curve and concentrations of aroma-active and key off-flavor compounds in TW.

No.	Key Flavor Compounds ^a^	FD	Quantitative Ion (m/z)	Standard Curve	*R* ^2 b^	Concentration (μg/L) ^c^	Recovery (%)	RSD (%) ^d^
1	(*E*)-2-octenal *	>81	108.2, 83.1, 70.1	y = 0.6813x + 0.1175	0.9995	654.47	126	4.94
2	(*E,Z*)-2,6-nonadienal *	>81	109.1, 70.1, 67.1	y = 0.8418x + 0.0274	0.9934	459.54	112	4.86
3	(*E*)-2-heptenal ^#^	81	112.0, 83.1, 70.1	y = 0.2955x + 0.0183	0.9959	1090.23	111	6.08
4	Decanal ^#^	81	209.0, 193.0, 70.1	y = 0.2869x - 0.0088	0.9921	93.37	99	0.23
5	Octanol ^#^	81	96.9, 84.1, 69.0	y = 0.5663x + 0.0091	0.9992	259.1	82	6.04
6	(*E,Z*)-2,6-nonadienol *	81	122.2, 81.0, 69.1	y = 0.4322x - 0.0065	0.9928	825.18	87	7.83
7	diisopropyl disulfide ^#^	81	150.1, 108.0, 66.0	y = 2.2337x + 0.6887	0.9974	2122.17	72	0.99
8	Hexanol ^#^	81	134.1, 119.0, 56.2	y = 0.8208x + 0.0072	0.9985	18.68	98	8.97
9	(*E*)-2-nonenal *	27	122.0, 83.1, 70.1	y = 0.8483x + 0.1171	0.9919	597.69	114	5.79
10	(*E*)-2-decenal ^#^	27	136.0, 83.1, 70.1	y = 0.6988x + 0.1383	0.9929	293.72	121	7.34
11	Nonanol *	27	182.0, 70.1, 50.1	y = 1.2630x + 0.0130	0.9983	17.77	77	8.71
12	(*E*)-2-octenol ^#^	3	146.8, 81.1, 57.2	y = 0.2583x + 0.0231	0.9939	16.73	109	0

a “*” means they were the aroma-active compounds; “#” means they were the key off-flavor compounds; b correlation coefficient of standard curve; c average concentration of triplicate experiments; d relative standard deviation.

**Table 4:** Odor activity values (OAV) of aroma-active and key off-flavor compounds.

No.	Key Flavor Compounds	Threshold (μg/L) ^a^	OAV
7	diisopropyl disulfide	0.1 [37]	21,222
9	(*E*)-2-nonenal	0.19 [38]	3146
2	(*E,Z*)-2,6-nonadienal	0.7 [38]	656
6	(*E,Z*)-2,6-nonadienol	1.3 [38]	635
10	(*E*)-2-decenal	1 [39]	294
1	(*E*)-2-octenal	3 [39]	218
4	decanal	0.9 [40]	104
3	(*E*)-2-heptenal	13 [41]	84
11	nonanol	2 [40]	9
5	octanol	110 [39]	2
8	hexanol	500 [42]	<1
12	(*E*)-2-octenol	40 [42]	<1

**Table 5:** Omission experiments from complete recombinate.

No.	Compounds Omitted from the Complete Recombinate ^a^	*n* ^b^	Significance ^c^
1	(*E*)-2-heptenal	4	
2	decanal	5	
3	octanol	10	***
4	diisopropyl disulfide	8	*
5	hexanol	9	**
6	(*E*)-2-decenal	8	**
7	(*E*)-2-octenol	8	*

a Reference standards for preliminary determination of seven key off-flavor compounds; b Number of correct judgments from 12 sensory panelists who evaluated the flavor difference by the triangle test; c “*”, significant (*p* ≤ 0.05); “**”, highly significant (*p* ≤ 0.01); “***”, very highly significant (*p* ≤ 0.001).
